# Why is violence high and persistent in deprived communities? A formal model

**DOI:** 10.1098/rspb.2022.2095

**Published:** 2023-02-22

**Authors:** Benoît de Courson, Willem E. Frankenhuis, Daniel Nettle, Jean-Louis van Gelder

**Affiliations:** ^1^ Max Planck Institute for the Study of Crime, Security and Law, Freiburg im Breisgau, Germany; ^2^ Institute for Education and Child Studies, Faculty of Social and Behavioural Sciences, Leiden University, Leiden, The Netherlands; ^3^ Department of Psychology, Utrecht University, Utrecht, The Netherlands; ^4^ Institut Jean Nicod, Département d’études cognitives, École Normale Supérieure, Université PSL, EHESS, CNRS, Paris, France; ^5^ Population Health Sciences Institute, Newcastle upon Tyne, Newcastle University, UK

**Keywords:** aggression, economic inequality, poverty, formal modelling

## Abstract

There is massive variation in rates of violence across time and space. These rates are positively associated with economic deprivation and inequality. They also tend to display a degree of local persistence, or ‘enduring neighbourhood effects’. Here, we identify a single mechanism that can produce all three observations. We formalize it in a mathematical model, which specifies how individual-level processes generate the population-level patterns. Our model assumes that agents try to keep their level of resources above a ‘desperation threshold’, to reflect the intuitive notion that one of people's priorities is to always meet their basic needs. As shown in previous work, being below the threshold makes risky actions, such as property crime, beneficial. We simulate populations with heterogeneous levels of resources. When deprivation or inequality is high, there are more desperate individuals, hence a higher risk of exploitation. It then becomes advantageous to use violence, to send a ‘toughness signal’ to exploiters. For intermediate levels of poverty, the system is bistable and we observe *hysteresis*: populations can be violent because they were deprived or unequal in the past, even after conditions improve. We discuss implications of our findings for policy and interventions aimed at reducing violence.

## 1. Introduction

There is massive variation in neighbourhood levels of interpersonal violence across time [1] and space [2]. For instance, homicide rates varied more than 100-fold between Chicago neighbourhoods in the 1988–1993 period [3]. This variation is partially explained by macro-level factors [4], such as economic conditions, poverty and inequality in particular [3–8], with extremely disadvantaged neighbourhoods having an unusually high level of violent crime [9]. Violence is also a locally persistent phenomenon, subject to *enduring neighbourhood effects* [10]. That is, violent places tend to remain violent to some degree, despite continuous population flux and even when economic conditions improve [10–12].

Different bodies of research have offered explanations of these observations at different scales. At the population level, several criminological theories view poverty as interfering with the normal functioning of a community, creating ‘strain’ [13,14]. Other criminological theories propose that poverty renders neighbourhoods deficient in social organization [12] or social efficacy [10], promoting violence and crime. A separate body of work has focused on the role of inequality, arguing that inequality creates a fiercer competition for symbolic and material resources, resulting in higher violence [5,15–17].

At the individual and psychological levels, several authors have pointed to the role of time preferences [3,18]. Poverty [19,20] and inequality [3] are related to poorer future prospects, including higher mortality and morbidity rates. This can result in a sense of futurelessness, which in turn leads to steep future discounting and choosing actions that can lead to immediate payoffs, such as crime [18,21]. Yet, other authors report that violence is a signal that serves to communicate a toughness reputation and avoid being victimized. This idea has been proposed independently in a variety of fields: ethnography [22,23], sociology [24], cultural psychology [25] and evolutionary psychology [26]. On this view, violence also has long-term benefits and does not necessarily qualify as a short-term strategy. Therefore, time preferences alone cannot explain the social gradient of violence.

In this article, we show that a single mechanism is able to generate all three key observations: violence is higher in deprived or unequal populations, varies considerably between populations, and can persist in a community despite economic improvement. Whereas the theories mentioned above are stated verbally, we articulate our explanation in a formal model. This serves two purposes. The first is to prove that the mechanism we propose is indeed able to reproduce the empirical observations at a qualitative level (i.e. the model's *generative sufficiency* [27]). If it is, our model can be considered as a valid *candidate explanation* [27,28]. Second, formalization eliminates the ambiguity inherent in natural language [29] and compels the provision of a fully explicit mechanism [30,31]. In particular, the process of formalization forces the specification of how interactions at the individual level produce group-level outcomes, which in turn shape individual behaviour. Thus, our approach aligns with the key aim of criminology and the social sciences generally to integrate micro- and macro-level processes [32,33]. This model uses ideas from complexity science. In that field, several models have studied crime (for a review, see [34])—yet without engaging with the role of material circumstances.

That violence is a social problem [35] does not imply that it reflects a dysfunction at the individual level. Here, we propose that violence is a ‘contextually appropriate response’, meaning it can be understood as a response to the costs and benefits associated with living in a particular context—as opposed to, for instance, a psychopathology or failure of willpower [36]. In our model, individuals make decisions based on their level of resources and other individuals’ behaviours. This game-theoretical feature creates the possibility that optimal strategies at the individual level produce suboptimal outcomes for the population, such as high rates of community violence, analogously to a ‘tragedy of the commons’ [37,38].

Analysing violence as a contextually appropriate response requires first the specification of its possible costs and benefits for an individual. Violence is commonly assumed—for instance, in the classic Hawk–Dove model [39]—to allow an agent to take a resource by force while facing a physical risk. In addition to these assumptions, we incorporate the idea that violence has reputational consequences as a ‘toughness signal’ [22–26], reducing the probability of being exploited. We thus focus on interpersonal violence involving physical harm to others for instrumental or reputational motives, rather than other forms of violence, like self-harm, child abuse, intimate partner violence or warfare. Our question then becomes ‘why would it be more appropriate to send such signals in deprived or unequal neighbourhoods?’. These neighbourhoods can be characterized as having a larger number of ‘desperate individuals’, without enough resources to meet their basic needs. They can be compared to drowning individuals, who would do anything to try to get their head out of the water, including dragging others down. Despite the high potential costs [40], exploiting others can be the most direct way to get resources quickly and jump back ‘above water’. We argue that the risk of being exploited by desperate individuals, in turn, triggers an incentive to send toughness signals (i.e. violent displays) among non-desperate individuals, to protect themselves from being targeted.

To formalize this intuition, we make two main assumptions. To represent the ‘signal effect’ of violence, we assume that being violent makes one less likely to be selected as a target of property crime. To represent financial desperation, we assume that agents are defined by a dynamic level of resources and have a ‘desperation threshold’, below which it is harmful to fall. In other words, agents are trying to always meet their basic needs and keep their head above water. The assumption of a threshold is a theoretically innovative idea in the social sciences [40] that we believe to be reasonable. It is inspired by optimal foraging theory in ethology [41], where models commonly include a ‘starvation threshold’ below which fitness rapidly declines. In humans, there are ethnographic descriptions of such thresholds [42–44], involving both physiological needs (e.g. hunger) and social needs (e.g. being respected), thus more generally the ability to meet basic needs. In the Philadelphia community he studied, Du Bois [44] described the poorest individuals as a ‘submerged tenth’, who are more likely to engage in dangerous actions. Scott [43] later observed among South Asian farmers a ‘subsistence crisis level—perhaps a ‘danger zone’ rather than a ‘level’ would be more accurate […] a threshold below which the qualitative deterioration in subsistence, security and status is massive and painful’ (p. 17). Experimental games have found that humans adjust their levels of risk taking in response to such thresholds [45,46], including by stealing resources from other participants [47]. Here, we assess the explanatory power of the desperation threshold for the socio-economic gradient of violence.

## 2. Model

The model is more thoroughly described in the electronic supplementary materials. The Python code can be found at https://github.com/regicid/model.

### (a) Structure of the model

Our model combines an individual-level optimal decision model and a population structure. The individual-level component is a state-dependent optimization algorithm, implemented by stochastic dynamic programming [48,49]. Agents are defined by a dynamic ‘state variable’ that represents their level of resources. It is affected by the agent's actions and by random fluctuations, following an AR(1) process with autocorrelation *r*. The model assumes a ‘desperation threshold’, a value below which agents are heavily penalized. They have access to several strategies, defined by probabilistic consequences on their level of resources, conditional on other agents’ strategies. Agents choose the strategy associated with the highest ‘fitness’, a *maximand* criterion that represents the agents’ goal. We allow the chosen strategy to depend on (i) the agent's level of resources and (ii) the frequencies of strategies in the population. In other words, agents pick the strategy which is optimal for their current level of resources and social environment.

The individual-level model identifies the optimal action an individual should choose for any given distribution of strategies in the surrounding population. It is not sufficient for revealing how that distribution will evolve. To address this population-level question, we simulate large populations of interacting agents with different levels of resources. By varying the initial distribution of those resource levels—in particular their mean and variance, representing economic affluence and inequality, respectively—we can test how the economic context affects the level of violence. We run the simulations until the system reaches a stable equilibrium. We also vary the initial distributions of strategies to examine the possibility of *hysteresis*, the dependence of the outcome on the initial conditions of the system.

### (b) Individual strategies and fitness

At each time step, agents can choose between three strategies: ‘exploitation’, ‘violence’ and ‘submission’. Exploitation represents property crime. When exploiting, an agent tries to take *β* resources from another agent. This strategy entails two potential costs. First, the exploiter might be caught and sanctioned with a probability *γ*, costing *π* units of resources (see table 1 for a summary of the parameters). This reflects exogenous social control, such as policing. We set the probabilities and magnitude of these costs (*β*, *γ* and *π*) such that the expected payoff of stealing is always negative, ensuring exploitation stays on average a bad decision. Second, the target agent may react violently: agents either fight (‘violence’ strategy) or not do so (submission). ‘Exploiters’ are also assumed to react violently to exploitation, but henceforth we use ‘violent’ to describe an agent who plays the violent strategy but does not exploit. The fight's winner, selected by a coin toss, obtains or keeps the disputed *β* resources. The loser pays a proportionate fitness cost *λ*, described in the next subsection. We also assume that violent agents sometimes enter unnecessary fights: with probability *m* , they attack a non-exploiter agent by mistake. This triggers a fight if the other individual is also violent.

**Table 1.** Notation summary.

symbol	meaning	typical value or range
*n*	population size	10^5^
*µ*	mean resource level	(5, 25)
*σ*	variance of resource levels	(4, 10)
*r*	resource levels autocorrelation	0.99
*n*	number of possible targets	(1, 50)
*β*	exploitation resource stake	10
*π*	resource cost of punishment	20
*γ*	probability of punishment	1/3
*m*	probability of violent mistake	(0.01, 0.3)
*ω*	fitness cost of being below threshold	(0.01, 0.3)
*λ*	lost fight fitness cost	(0.01, 0.3)

The violent strategy also sends a ‘toughness signal’: it is observable and confers a ‘toughness reputation’, which reduces the probability of being the target of exploitation at this round. We assume that choosing the violent strategy suffices to confer this reputation, even for an agent who has never fought. This is a simplifying assumption, that also guarantees the coherence of the model (see electronic supplementary materials). Exploiters choose their target out of a set of *n* randomly drawn agents, among which they prefer submissive targets over violent ones. Agents playing ‘violent’ at this round, therefore, have (if *n* > 1) a non-zero but lower probability of being victimized than agents playing ‘submissive’ (see electronic supplementary materials for details). The parameter *n* controls how much lower, and thus represents the toughness signal's efficiency: it has no effect if *n* = 1, whereas if *n* → ∞, then violent agents are never victimized as long as there is at least one submissive agent in the population. The ‘violent’ strategy is designed to represent forms of violence involving either a material or a reputational stake, our model thus focuses on these forms.

Agents choose the strategy that yields the highest expected ‘fitness’, defined as the resource level attained after T periods reduced by (i) a fraction *ω* for every period spent below the desperation threshold and (ii) a fraction *λ* for every lost fight, representing the risk of an injury when fighting. In other words, agents try to maintain their head above water financially while fighting as rarely as possible. Since we use a Stochastic Dynamic Programming algorithm, the decision takes into account not only the possibility of being below the desperation threshold immediately but also at any later point in the future.

### (c) Population simulations

We simulate populations of *n* = 10^5^ agents whose level of resources are drawn from a Gaussian distribution. At every time step, we let 10% of the population, randomly chosen, update their strategies. This way, the distribution of strategies in the population can evolve smoothly to an equilibrium rather than oscillating. When updating their strategies, agents take into account their level of resource and the current frequency of ‘exploitation’ and ‘violence’ in the population. For simplicity, we assume agents have perfect knowledge of others’ strategies. We iterate this process enough times for the distribution of strategies to stabilize.

We then compare the outcomes of the model for different values of the mean *µ* and the variance *σ* of the distribution of resources in the population, representing economic affluence and inequality, respectively. To investigate the possibility of *hysteresis*, we test if the outcome depends on the initial proportion of violent agents. In the electronic supplementary materials, we explore how the model is affected by varying the other parameters.

## 3. Results

### (a) Individual decisions

**Figure 1.**
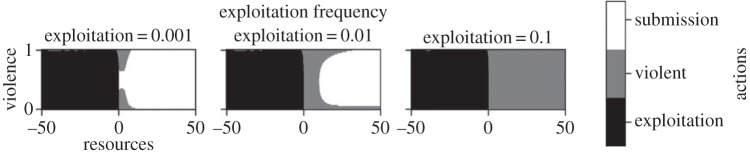
Optimal strategies depending on resource levels (*x*-axis), prevalence of violence (*y*-axis) and prevalence of stealing (panel). The central tick on the *x*-axis (0) represents the desperation threshold. Agents tend to exploit below the threshold and to be violent either when exploitation is frequent, when violence is very rare or when violence is frequent.

Figure 1 represents the optimal decisions depending on resource levels and the prevalence of exploitation and violence in the population. It is generally optimal to exploit when one is below the desperation threshold, confirming the previous finding that being underwater makes risk-taking contextually appropriate [40]. The area where agents exploit is roughly a square. This decision is thus virtually independent of the level of violence, even though exploitation is greatly disincentivized by the presence of violent agents. In a fully violent population, an exploiter will always have to fight, which halves its probability of success and reduces its ‘fitness’ in case of loss.

Above the threshold, agents should be violent in several cases. First, when exploitation is frequent (in figure 1, compare the right panel to the left). This is unsurprising: violence lowers the risk of being exploited, so the higher this risk is, the more agents should protect themselves. Second, when violence is very rare (in figure 1, at the bottom of the *y*-axis). A lone violent agent will never be exploited (as the exploiters will always be able to choose a submissive target instead) and never fight, as it never meets other violent agents. Therefore, being violent in a fully non-violent environment has benefits but no costs, and is favoured.

Agents should also choose violence when violence is very frequent (in figure 1, at the top of the *y*-axis). The more violence there is, the more exploitation is concentrated on the rare submissive agents. This incentivizes them to be violent, and thus violence begets violence. Finally, agents are prone to violence when close to the threshold. This is due to risk preferences: being violent reduces the risk of being exploited. Just as agents are risk-prone below the threshold because they have ‘nothing to lose’, they are risk-averse just above the threshold as they have ‘too much to lose’, like a person on the edge of a cliff. Intuitively, close-to-the-edge individuals choose to risk their health in an attempt to hang on to their resources.

### (b) Population simulations

**Figure 2.**
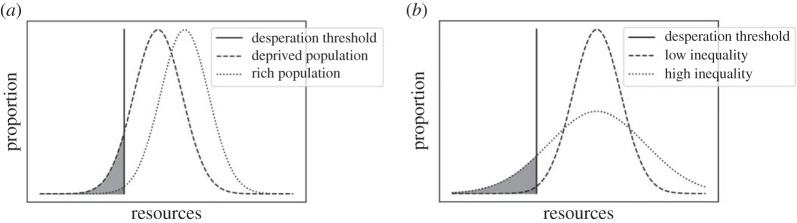
Effect of poverty (*a*) and inequality (*b*) on desperation rate. Either reducing the mean of the resource distribution or increasing its variance leads to a larger tail of individuals whose resources are below the desperation threshold.

At the population level, outcomes depend chiefly on the proportion of agents below the desperation threshold, the ‘desperation rate’. This rate determines the number of exploiters, which in turn determines the number of violent agents (figure 1). Both poverty (low mean level of resources *µ*) and inequality (high variance *σ*) increase the desperation rate: the lower or the broader the distribution, the larger the left tail consisting of desperate agents (figure 2).

**Figure 3.**
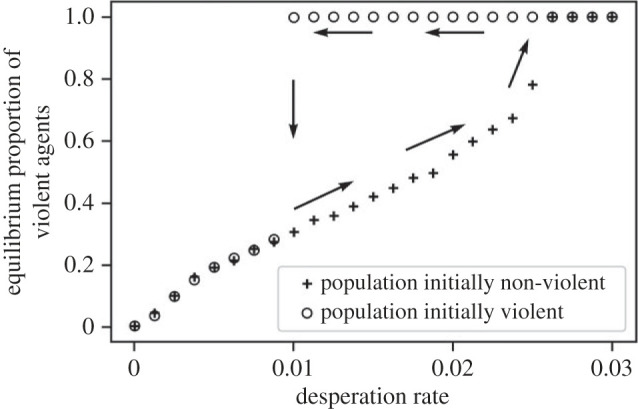
Proportion of agents playing ‘violent’ or ‘exploiting’ at equilibrium against the desperation rate. The crosses show the data for populations initialized with no violent strategies, and the circles populations initialized with all violent agents. We cut the *x*-axis when both curves reach 1 (beyond 0.03, crosses and circles stay at 1). The arrows illustrate the *hysteresis* loop: if a population's desperation rate increases from 0 to 0.04, it would follow the lower branch, but if it decreases from 0.04 to 0, it would follow the upper branch.

The higher the proportion of desperate agents, the higher the prevalence of violence at equilibrium (figure 3). In our model, therefore, both poverty (*µ*) and inequality (*σ*) increase violence. However, the relationship of the desperation rate to the equilibrium frequency of violence depends on how we initialize the strategies. If we begin with no violent agents in the population, we reach the equilibrium frequencies shown with the crosses on figure 3. We observe an inverse s-shaped function. For low values of desperation rate, we have a concave relation: the violent strategy gets costlier as it spreads, dampening the increase. For high values, we observe a convex relation revealing a positive feedback effect: exploitation is deflected onto the submissive agents, and violence begets violence. In the electronic supplementary materials, we show that this result is qualitatively robust to changes in the exogenous parameters values.

Here, it must be noted that inequality only plays a role by increasing the amount of desperate agents. In other words, inequality increases violence through absolute poverty, not relative poverty. Concretely, enriching the rich without impoverishing the poor would not increase violence. It must however be noted that we assumed the desperation threshold to be fixed and independent on the average level of resource *µ*, which, by construction, precludes the role of relative deprivation. We explore this limitation in the Discussion.

### (c) Multiple equilibria and *hysteresis*

**Figure 4.**
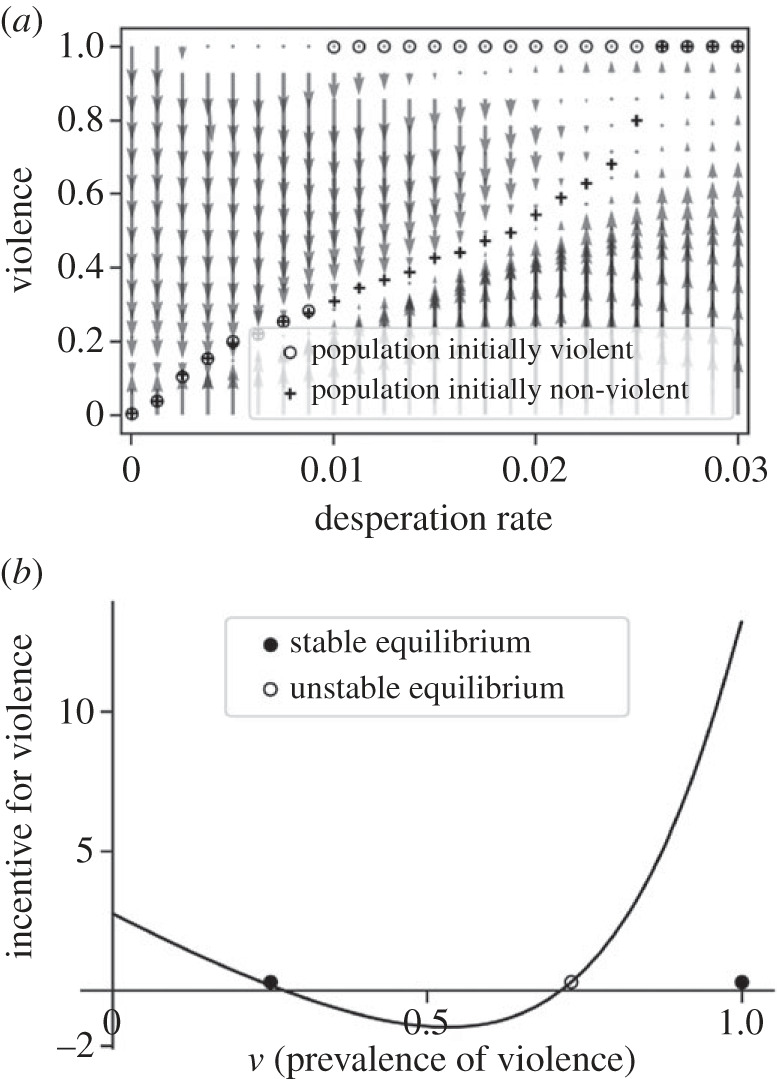
(*a*) Vector field representation of the model results. We obtain the vector field numerically, by initializing the population with a certain proportion of violent agents and a certain desperation rate. We let agents update their strategies and the arrows represent how much the proportion of violent agents has changed. For intermediate desperation rates, the vector field reveals a bistable system, explained by (*b*). (*b*) Incentives for violence, defined as the mean difference in payoffs between the violent and submissive strategies, obtained with a fixed desperation rate, varying the prevalence of violence. The incentive for violence first decreases as the risk of actually fighting increases, then explodes as the costs of exploitation concentrate on the rare non-violent agents.

If we initialize the simulation with only violent agents, the population moves to a completely violent configuration in a large range of the desperation rate range (figure 3, circles). Here, the system has two distinct stable equilibria. Which of these is reached depends on where the system comes from. We observe a *hysteresis* loop: if the desperation rate (here exogenous) increases from 0 to 0.03, the system follows the lower branch, but if it decreases from 0.03 to 0, it follows the upper branch and remains in a fully violent configuration for a long time. Thus, two equally deprived or unequal communities can have vastly different levels of violence for historical reasons, with high desperation in the past producing a persisting high violence. We can visualize this bistability using a vector field (figure 4*a*). The upper equilibria have small basins of attraction, and will therefore not be reached unless a very large share of the population is violent. However, figure 4*b* shows that the incentive to be violent soars as the prevalence of violence goes to 1, as rare non-violent agents concentrate exploitation costs. Thus, even though a few non-violent agents would suffice to reach the basin of attraction of the lower equilibrium, the model predicts these equilibria to be stable and robust to small changes in the parameters. In the electronic supplementary materials, we show that the *hysteresis* effect holds if the parameters *r* and *n* are high enough, that is, if toughness signals are efficient enough and the experience of desperation sufficiently persistent.

## 4. Discussion

Violence rates vary considerably throughout time and space, in association with economic deprivation and inequality, and scan have a persistent character. We have shown that a single mechanism can account for these three empirical observations. We therefore offer a *candidate explanation*, proving that some conditions are *sufficient* to generate a particular phenomenon. The innovation in this work resides in demonstrating the consequences that flow from making two original assumptions, namely (i) that agents have a desperation threshold and (ii) that violence sends a ‘toughness signal’ that reduces the risk of being exploited. Assumption (i) has been explored in a previous model to explain property crime, but not in relation with violence. Assumption (ii) has been explored earlier in game theoretical models of conflict [50–52], but not yet in relation to deprivation and inequality.

### (a) Desperation triggers high violence

The assumption that agents have a desperation threshold has important consequences. First, it triggers risk-proneness below the threshold [40]. Intuitively, desperate individuals have ‘little to lose’: if their gamble succeeds, they lift their head above water, and if it fails, it makes little difference. As we assume that stealing entails the highest variance, ‘desperate’ agents are likely to exploit. This result holds as long as individuals possess such a threshold. The ‘threshold’ idea can have several interpretations, starvation being the most obvious, but perhaps not the most relevant for the inhabitants of industrialized countries.

Therefore, a larger proportion of desperate agents—as a consequence of either poverty or inequality (figure 2)—leads to more violence (figure 1), as non-desperate individuals try to reduce the risk of exploitation. The exploitation that stems from being below a desperation threshold is largely insensitive to the magnitude of punishment [40], in line with empirical evidence [53]. As individuals scramble to get back above the threshold, desperate individuals care more about the maximum payoff than the expected payoff of their strategy. In our model, this means that desperate agents continue to steal even when facing a high risk of violence. As a consequence, violence acts as a ‘deflector’ rather than a deterrent: it will not prevent exploitation, but might make the offender shift to a different, non-violent target.

This ‘deflector’ property of violence fundamentally influences the results of the model. In conventional rational choice models [54], violence deters stealing by increasing its potential cost. In such models, violence acts as a ‘thermostat’, dampening variation in rates of property crime—a sort of homeostasis. This prediction is at odds with empirical evidence showing massive variation in rates of violence across space and time [2]. Instead our model predicts, due to the desperation threshold, that high rates of property crime can persist despite high rates of violence, which is more consistent with the empirical record [55].

### (b) Why is violence persistent?

Despite the common intuition that ‘violence begets violence’, standard game theory models actually predict the reverse. For instance, the hawk–dove model [56] finds violence to be a negatively frequency-dependent strategy: as violence becomes more common, a violent individual is more likely to meet another violent individual and to get into a costly fight. Put differently, every increase in violence diminishes its appeal, which stifles its spread. For that reason, the hawk–dove model predicts that the population only reaches a pure equilibrium where all individuals adopt a violent strategy when the cost of losing a fight is smaller than the cost of the resource at play [56]. Again, this suggests that violence should display little variation between communities: the costs and benefits might vary, but the negative frequency dependence should homogenize violence rates. This mechanism—violence becoming more costly as it spreads—also operates in our model. However, the assumption that violence confers a ‘toughness reputation’ counteracts this dynamic. As being violent deflects property crime on non-violent agents, the spread of violence also makes non-violence more costly. The frequency dependence reverses for high enough levels of violence and turns into a positive feedback, whereby violence actually begets violence. Which one of these dominates depends heavily on the prevalence of exploitation, which in turn depends on the proportion of desperate individuals. Thus, our model recovers the potential for rates of violence to vary sharply in a way that depends notably on socioeconomic deprivation.

In some regions of the parameter space, high- and low-violence configurations can simultaneously be stable equilibria in our model. Depending on the starting point, the population can end up in either of the two equilibria: if violence is low, then it stays low due to the negative frequency dependence; if violence is high, it stays high due to the positive feedback. If violence is rare, it is not worth sending a violent signal as the risk of victimization is diluted in the population, whereas if the vast majority of individuals are violent, a rare non-violent agent will bear the brunt of victimization and suffer untenable costs. To understand this result more intuitively, one can think of bike locks, which play a protective role analogous to violence in our model. When leaving your bike among dozens of unlocked bikes, it may not be necessary to lock it, as the risk of it being stolen is diluted among all the bikes. However, if all the bikes around are locked, a stealer passing by would likely steal the only unlocked bike. Similarly, the risk of being exploited might be low enough in the low-violence equilibrium for the cost of violence to be too high to incur. In the high-violence equilibrium, however, any submissive deviant will inevitably concentrate the risks of exploitation.

This situation is analogous to coordination games, where positive frequency dependence generates multiple equilibria—for instance left- and right-hand driving. But whereas collective wellbeing is roughly equal whether cars drive left- or right-wing, in our case, settling in a high- or low-violence equilibrium is very consequential. An earlier evolutionary game theory model of toughness signals also reports the possible coexistence of two equilibria with very different levels of aggression [52]. The authors conclude that a population is unlikely to persist in the high-violence equilibrium, as mean fitness is lower than in the low-violence one. Our model does not include an equilibrium selection process. But in the human case and for the relatively short timescales we are interested in, extending only to a few generations, it seems plausible that a community could be trapped in such a detrimental equilibrium, and that such an equilibrium can therefore be empirically relevant.

This bistability generates a *hysteresis* effect: violence rates do not only depend on the current economic conditions, but also on their history. Concretely, a neighbourhood can be more violent than an equally rich neighbourhood because it was poorer and more violent in the past. Thus, violence can persist despite some economic improvement. This result aligns with the ‘enduring neighbourhood effect’ [10] of violence, and thus offers an alternative explanation for it—not necessarily incompatible with the prevailing approach, the social efficacy theory [57].

### (c) Relative poverty or absolute poverty?

In our model, poverty and inequality only increase violence through the proportion of desperate agents. Thus, the effect is driven only by absolute poverty, and not by relative poverty. Concretely, our model predicts that making the richest individuals richer with no impact on the poorest does not increase violence. This might seem at odds with several empirical findings [3,5,8,58] and theories [15,21,59,60]. For instance, comparing the neighbourhoods of Chicago, Wilson & Daly [3] report that when controlling for economic inequality, median household income did not predict homicide rate in Chicago neighbourhoods. However, our model's prediction is a consequence of our assumption that the desperation threshold is exogenously fixed across communities. Instead, the threshold itself may increase with affluence and with inequality. For example, the existence of inequality has been found experimentally to increase individuals’ ‘perceived needs’ [61]. In our model, if the threshold was assumed to be proportional to the median resource level (as the poverty line is defined in the European Union), then any change in poverty through the parameter *µ* would affect equally both agents’ level of resources and the threshold, without consequence. In this setting, the level of violence would only depend on inequality (*σ*). One could also imagine that an exploited agent loses a proportion of his wealth instead of a fixed amount of resources. In this setting, the presence of very well-off individuals could act as an incentive for exploitation, which could in turn create a stronger need for protection among these individuals.

### (d) Future directions

Our model analyses violence as a binary decision, where choosing ‘violence’ essentially means being ready to fight when exploited, which yields a ‘toughness reputation’, known by all of the other agents. This assumption is restrictive in two respects that we could explore in the future. First, we could allow agents to fine-tune their level of violence. This would require specifying how the fitness costs of violence relate to the level of violence and how exploiters choose their targets in this new context. It would be interesting to test, for instance, whether the *hysteresis* effect holds in this case, and to observe if the insensitivity to deterrence induced by the desperation threshold triggers arms race dynamics through an ‘inflation of toughness’.

Second, our assumptions implicitly entail that an agent simply needs to be ready to fight so that every other agent treats him as tough, even if she has never fought. In our simulations, the majority of violent agents reap the benefits of violence without actually paying the costs, as fights rarely occur. We chose here to directly specify how the signal changes the receiver's behaviour, in order to focus on the population dynamics. An alternative assumption would be to only attribute a toughness reputation to an agent after she actually fought. This would make the model more complex, but would enable it to study another question: under what conditions should an agent be violent without material necessity, for pure reputational reasons? This could shed light on Anderson's observation that, in the deprived communities he studies, ‘there are always people around looking for a fight’, a situation he attributes to ‘campaigning for respect’ [22].

Finally, our model also generates novel testable predictions and can guide empirical data collection. For instance, we predict individuals to be violent when they live in a deprived neighbourhood, whether they are themselves materially deprived or not (figure 1). This suggests that an individual's attitude toward violence would be better predicted by the economic situation of their neighbourhood, rather than by their own personal socio-economic status, whereas the reverse would be true for property crime. It is reminiscent of Anderson's observation that in deprived neighbourhoods, relatively better-off ‘decent families’ reluctantly teach the ‘code of the streets’ to their children, as they consider a toughness reputation as necessary to navigate this social environment [22]. Cohen & Nisbett also found that geographical origin (North or South) determined whether individuals endorsed ‘culture of honour’ values, regardless of their current circumstances [25].

### (e) Implications for policy and intervention

We discuss implications of our model for public policy and intervention. First, our model predicts that helping the poorest individuals to get back above the threshold of economic deprivation can have a ripple effect. Not only would such help reduce the probability that desperate individuals choose to steal, violence would also diminish among the rest of the population, for whom there has been no change in resource levels. In our model, the dire poverty of some has an emergent effect on the population: in a world where some individuals are ready to do anything to get their head above water, everyone must take protective steps like violence. By eliminating desperation, one might then improve the welfare of all members of the group, even those who do not benefit from the policy.

The second implication is related to the *hysteresis* effect our model produces. For a community trapped in a high violence equilibrium (figure 3, upper branch), the effect of an economic intervention would be a step function (figure 3, top arrows). Minor interventions would have no effect, then at some point, a sufficiently large intervention would cause a phase transition to a low violence equilibrium, and have a massive impact (figure 3, vertical downward pointing arrow). Similarly, interventions on violence (i.e. lowering violence without changing the desperation rate) would need to be sufficiently large to have an effect. In figure 4*b*, it would be necessary to pass the unstable equilibrium to reach the basin of attraction of the low-violence equilibrium, otherwise violence goes back to maximum when the intervention ends. Therefore, our model suggests a nonlinear relationship between intervention magnitude and resulting change. It also calls into question the use of linear models to test the efficiency of these interventions. An empirical researcher might for instance find a null effect of economic support on violence if she only looks at a limited intervention, whereas more of this same intervention could have a tremendous positive impact.

To conclude, we presented a simple model combining individual optimal decisions and population simulations. We make two original assumptions: agents have a desperation threshold, and violence serves as a ‘toughness signal’. We show that their combination is able to explain three cornerstone empirical findings: the large variation of violence rates between neighbourhoods or communities; the effect of poverty and inequality on violence; and the persistence of violence across time.

## Data Availability

The Python code of the model and of the figures can be found at https://github.com/regicid/model_deprivation_violence. An online notebook to manipulate the model online without installing Python is available at: https://colab.research.google.com/drive/1wf3KBd95YO_WTluGztaR-8l-1zOD-e0o?usp=sharing. The data are provided in the electronic supplementary material [62].
